# Classification and Segmentation of Nanoparticle Diffusion Trajectories in Cellular Micro Environments

**DOI:** 10.1371/journal.pone.0170165

**Published:** 2017-01-20

**Authors:** Thorsten Wagner, Alexandra Kroll, Chandrashekara R. Haramagatti, Hans-Gerd Lipinski, Martin Wiemann

**Affiliations:** 1 Biomedical Imaging Group, Department of Informatics, University of Applied Sciences and Arts Dortmund, Dortmund, Germany; 2 EAWAG, Swiss Federal Institute of Aquatic Science and Technology, Dübendorf, Switzerland; 3 Experimental Physics IV and Bayreuth Insitute for Macromolecular Research, University of Bayreuth, Bayreuth, Germany; 4 IBE R&D gGmbH Institute for Lung Health, Münster, Germany; Brandeis University, UNITED STATES

## Abstract

Darkfield and confocal laser scanning microscopy both allow for a simultaneous observation of live cells and single nanoparticles. Accordingly, a characterization of nanoparticle uptake and intracellular mobility appears possible within living cells. Single particle tracking allows to measure the size of a diffusing particle close to a cell. However, within the more complex system of a cell’s cytoplasm normal, confined or anomalous diffusion together with directed motion may occur. In this work we present a method to automatically classify and segment single trajectories into their respective motion types. Single trajectories were found to contain more than one motion type. We have trained a random forest with 9 different features. The average error over all motion types for synthetic trajectories was 7.2%. The software was successfully applied to trajectories of positive controls for normal- and constrained diffusion. Trajectories captured by nanoparticle tracking analysis served as positive control for normal diffusion. Nanoparticles inserted into a diblock copolymer membrane was used to generate constrained diffusion. Finally we segmented trajectories of diffusing (nano-)particles in V79 cells captured with both darkfield- and confocal laser scanning microscopy. The software called “TraJClassifier” is freely available as ImageJ/Fiji plugin via https://git.io/v6uz2.

## Introduction

Transport processes of particulate structures inside cells are of pivotal importance for many cellular functions. The way how small objects move at the cell boundary may provide insight into mechanical properties of the local surroundings [1], and can unravel nanoparticle (NP) or even protein cell entry mechanisms [2–4]. In all these cases, single objects need to be imaged and their trajectories carefully analyzed. Basically, particle movements can be classified into four basic motion types: normal diffusion (ND), anomalous diffusion (AD), confined diffusion (CD) or directed motion (DM). ND takes place when particle movements occur completely unrestricted. DM is an active process and may become evident when small corpuscles such as vesicles are tansported by molecular machines along microtubules [5, 6]. CD is observable for trapped particles or particles whose free diffusion is confined by cytoskeletal elements [7]. The origin of AD is commonly traced back to the macromolecular crowding in the interior of cells, but its precise nature is still under discussion [8].

Arcizet et al. [9] classified particle trajectories in active and passive tracks based on the exponent of a fitted power distribution, and on the standard deviation of the angle correlation function. By applying their method to sub-trajectories using a sliding window the method allows distinguishing for multiple passive or active parts in a single trajectory. Huet et al. [10] calculated the diffusion coefficient, the curvature of the mean squared displacement curve, and the asymmetry of the trajectory. By using six different thresholds they classified the trajectories into constrained, directed and stalled motion categories. This approach could also be applied to sub-trajectories using a sliding window. However, both methods have in common that they classifiy only a subset of the four basic motion types, namely active and passive motion for Arcizet’s approach and confined diffusion, active motion and not moving particles for Huet’s approach. In another approach used by Suh et al. [11] only the so called “Relative Change” (RC) was evaluated, which was defined as the ratio of the calculated diffusion coefficient and a reference diffusion coefficient. The RC value was evaluated for two different time scales and classified into the categories diffusive, subdiffusion and active using confidence intervals of the RC value for normal diffusion. Unfortunately, the confidence interval has to be estimated for each track length which complicates the general application of the method. Furthermore, the approach does not allow a local analysis by a sliding window. Monnier and co-workers [7] used a Bayesian approach and distinguished seven different diffusion models. However, their method requires to choose between predefined probabilities which are associated with each diffusion model. Furthermore the performance decreases in case of heterogeneous modes of particle diffusion.

Altogether, the methods described above need extensive configuration, do not cover the analysis of all basic motion types, or have practical drawbacks. Recently we have reported first results obtained with a new method which classifies normal diffusion, subdiffusion and directed motion using a random forests approach trained by three features which were estimated for simulated trajectories [12]. However, the approach was neither applicable to confined diffusion nor to local analysis. Also it did not consider “localization noise” of particle movement. To further develop the approach, here we extend the method to all four basic motion types and also included a local analysis approach with a sliding window. We will give a brief introduction into basic motion models and how to obtain simulation data, since simulated trajectories had to be used to train a Random Forests classification model using nine different features. Furthermore we will give advice on parameter selection and investigate the working range by a sensitivity analysis. The refined method was applied to videos of diffusing particles captured by nanoparticle tracking analysis (NTA), to videos of particles in micron-sized confinements [13], and to videos of particles inside and outside of live cultured lung fibroblasts (V79). To view particles in cells, darkfield microscopy (DFM) and confocal laser scanning microscopy (CLSM) operated in the reflection mode were chosen. These studies on particle movements in cellular confinements are challenging as they offer heterogeneous particle trajectories different from free diffusion and, therefore, demand a validation of the program.

## Materials and Methods

This section describes the supported motion models. We will first give a description how motion types were simulated to obtain synthetic trajectories. Then the experimental setup is described for extracting trajectories from biological and non-biological systems. Eventually, a brief introduction into the random forest classification algorithm is given, followed by an overview of features used to train the classifier and to characterize the trajectories.

### General definitions

This section gives some general definitions of terms which are used throughout this article. A trajectory is a number of N consecutive two dimensional positions of a particle ***x***_***i***_ = (*x*_*i*_, *y*_*i*_) recorded with a constant time interval Δ*t* over a period of time *T* = (*N*−1)Δ*t*. A change in position from ***x***_***i***_ to ***x***_***i*+1**_ is called a step. The step length is defined as the euclidean norm |***x***_***i***_ − ***x***_***i*+1**_|. If only a part of a trajectory ***x***_***i***_…***x***_***i*+1**_ is used we call it a sub-trajectory. The empirical mean squared displacement (MSD) function is defined as

$$\begin{array}{ccc} \left\langle r_{n}^{2}\right\rangle =\frac{1}{N-n}\sum_{i=1}^{N-n}{\left|x_{i+n}-x_{i}\right|}^{2} & , & n=1,\ldots ,N-1 \end{array}$$ (1)

A msd curve model for a specific motion type fitted through the data points could be used to derive characteristic parameters like the diffusion coefficient *D*.

### Motion models

As explained in the introduction, single particle tracking in live cells has disclosed several motion types. These motion types are commonly characterized by the shape of their MSD curve. According to Saxton [14], the MSD function of the four basic motion types are given by:

$$\langle r^{2}\left(n\right)\rangle =4Dn\Delta t\;\;\text{Normal}\;\text{diffusion}$$ (2)

$$\langle r^{2}\left(n\right)\rangle =4D{\left(n\Delta t\right)}^{\alpha }\;\;\text{Anomalous}\;\text{diffusion}$$ (3)

$$\langle r^{2}\left(n\right)\rangle =4Dn\Delta t+{\left(vn\Delta t\right)}^{2}\;\;\text{Directed}\;\text{motion}\;\text{with}\;\text{diffusion}$$ (4)

$$\langle r^{2}\left(n\right)\rangle ≃r_{c}^{2}\left[1-A_{1}exp\left(-4A_{2}Dn\Delta t/r_{c}^{2}\right)\right]\;\;\text{Confined}\;\text{diffusion}$$ (5)

In Eq (3), the value of the anomalous exponent is *α* < 1. The DM is described in Eq (4) where *v* is the velocity of the DM. Eq (5) embodies confined diffusion where *r*_*c*_ is the radius of the confinement and the constants *A*_1_ and *A*_2_ are characterizing the shape of the confinement. When generating synthetic trajectories their MSD curves should at least follow these models. How these motion types were simulated is described in the following sections.

### Simulations

**Fig 1:**
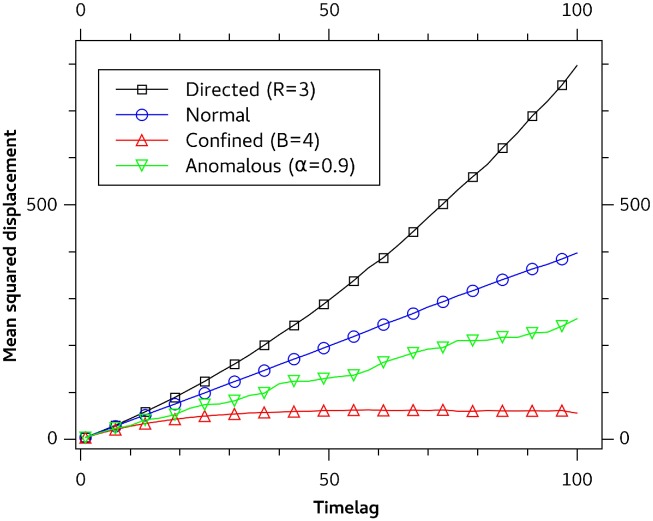
Mean squared displacment curve for simulated motion models. Mean squared displacment curves based on simulated trajectories for directed motion (black), normal diffusion (blue), anomalous diffusion (green) and confined diffusion (red).

In this section we will introduce the Monte Carlo simulations of the ND, CD, AD and DM. The MSD curves, calculated on the basis of these simulations do follow the motion models described by Eqs (2)–(5) as shown in Fig 1.

#### Normal diffusion

For the ND of a particle we randomly chose a start position, a random direction, then draw a random steplength from the steplength density *F*_*d*_ as described by Michalet [15]

$$\begin{array}{c} F_{d}\left(u\right)=\frac{2u}{4D\Delta t}e^{\frac{-u^{2}}{4D\Delta t}} \end{array}$$ (6)

where *u* is the absolute distance traveled by the particle during Δ*t*. Finally we added the displacement to the start position. For generating the next position, the last position was taken as start position and the procedure was repeated.

#### Confined diffusion

We assume that a particle starts from the center of 2-dimensional circular reflective boundary. To simulate a single step of a confined diffusion, 100 sub-steps were generated by drawing both random step length from Eq (6) with Δ*t*′ = Δ*t*/100 at random direction, and then update the latest position (of the sub-steps) if the distance from the center is smaller than the radius of the circular reflective boundary. Furthermore, we define a single dimensionless parameter B which characterizes as much a trajectory is restricted by its confinement. Simulations show that the area of the smallest rectangle of a trajectory enclosing a ND trajectory with N steps can be estimated by:

$$\begin{array}{c} A_{rect}\approx 4DN\Delta t \end{array}$$ (7)

The area of the smallest ellipse fitted by that rectangle is given by

$$\begin{array}{c} A_{ell}\approx \frac{\pi }{4}A_{rect}=\pi DN\Delta t \end{array}$$ (8)

We define the ratio *A*_*ell*_ of and the area of a confinement with radius r as the boundedness B:

$$\begin{array}{c} B=\frac{A_{ell}}{\pi r^{2}}=\frac{DN\Delta t}{r^{2}} \end{array}$$ (9)

This formula allows us to derive the confinement radius r for a given B. Furthermore the approach can be evaluated independent of the actual confinement radius and diffusion coefficient.

#### Anomalous diffusion

For generating trajectories representative for the (sub-)diffusive motion type we employed the Weierstrass-Mandelbrot function (WMF), which is a common high-quality method to simulate anomalous behavior [16–18]. The WMF is given by

$$\begin{array}{c} W\left(t\right)=\sum_{n=-\infty }^{\infty }\frac{cos\left(\phi_{n}\right)-cos\left(\gamma^{n}t^{*}+\phi_{n}\right)}{\gamma^{n\alpha /2}} \end{array}$$ (10)

with the anomalous exponent *α* < 1, t* = 2*πt*/*N*, $\gamma =\sqrt{\pi }$ and random phases *ϕ*_*n*_ being uniformly distributed between 0 and 2*π*. The sum is taken from n = -8 to n = +48 as described by Saxton [16]. For generating *N*′ steps, we take *N* ≫ *N*′ displacements *d*_*i*_ = *W*(*t*) − *W*(*t* − Δ*t*) to ensure an appropriate sampling and then use the first *N*′ displacements only to build the trajectory. The displacements for x- and y-direction are generated independently and scaled so that the MSD is $\langle r_{1}^{2}\rangle =4D\Delta t$.

#### Directed motion

DM was simulated by combining two trajectories of the same length: a ND trajectory generated according to the procedure described above is combined with a pure active motion trajectory. The active motion displacements *d*_*i*_ were calculated using the velocity *v* and the time lag Δ*t*: *d*_*i*_ = *v*Δ*t*. The direction was changed by an angular velocity of $\frac{\pi }{4}$ rad/s. After simulating both trajectories, their single positions are summed pairwise which give the final DM trajectory. How much a trajectory is influenced by a DM is characterized by the ratio R of squared velocity *v* of the active motion component and the diffusion coefficient *D* of the ND component:

$$\begin{array}{c} R=\frac{v^{2}T}{4D} \end{array}$$ (11)

where *T* is the time duration.

### Imaging equipment and data acquisition

The following subsections give an overview on how video data of diffusing particles in cell culture were captured and how data was handled. In later sections we will describe how trajectories were extracted from video files.

#### Optical recording of particles in vitals cells by dark field microscopy

Hamster fibroblasts (V79, cultured in DMEM supplemented with 10% fetal calf serum) were grown to ca. 50% confluence on a coverslip (20x20 mm^2^). To set up a live observation chamber, two strips of cover slip glass (5x20x0.17 mm^3^) glued on a glass slide served as spacers, onto which the cell laden cover slip was mounted upside down. The chamber was rinsed (0.1 ml/min) with Krebs Ringer solution containing 2 mM glucose (KRPG). The chamber was mounted onto the stage of an Olympus BX51 microscope heated to 37°C. The cell containing optical plane was illuminated with a CytoViva^*TM*^ darkfield oil condenser. To load cells with NP under optical control the chamber was perfused with a low concentration (5 *μ*g/ml) of PVP-coated gold NP (size: 50 nm, Nanocomposix) added to the superfusate. Image series were taken with a color CCD camera (PCO VGA) at a constant frame rate of 30 FPS.

#### Optical recording of particles in vitals cells by confocal laser scanning microscopy

CLSM was used to record NP movement inside V79 cells. V79 cells were seeded at 2x10^5^ cells/mL in 5 mL medium in glass-bottom petri dishes (CELLView No. 627860, Greiner, 35x10 mm, tissue culture treated). After 18 h, medium was exchanged and cells were imaged using a Leica SP5 setup equipped with a CO_2_ gassing and temperature control unit. Cells were thus maintained at the same conditions as during culturing. Samples were scanned with the 488 nm laser line and reflected light was detected from 483-493 nm along with the bright-field image (water immersion objective (63x/0.9)). Data was recorded continuously in a region of 41x41 *μ*m^2^ at a scan rate of 1400 Hz resulting in a frame duration of 94 ms; the pinhole was set to 600 *μ*m (5.778 *μ*m layer), image resolution was 256x256 pixel. After several minutes of recording, control medium was replaced carefully with medium containing 10 *μ*g/mL citrate-coated gold NP (size:50 nm, Nanocomposix). After imaging NP in the presence of V79 cells, samples were transferred back to regular culture conditions and imaged again after 24h as described above. Recorded data were exported in *.avi format without overlaying channels.

#### Nanoparticle tracking analysis of freely diffusing particles

Measuring the hydrodynamic diameter of NP by tracking light scattering NP in videos is an increasingly used technique [19–21]. NP suspensions are illuminated by a laser and an ensemble of light scattering particles is recorded over time by a CCD camera. The Brownian motion of each single NP is tracked using image analysis techniques. First, for each trajectory the mean squared displacement of the NP is estimated. Next, the corresponding diffusion coefficient is calculated and converted into the hydrodynamic diameter using the Stokes-Einstein equation. Finally, the calculated diameters are used to estimate the size distribution. Importantly, this method relies on low particle concentrations (ca. 5x10^8^ /ml) at which unrestrained particle diffusion takes place. As unrestricted diffusion is expectedly rare inside cells, we classified trajectories of videos of previous experiments with 100nm polystyrene NP (Kisker-Biotech, PPs-0.1) which were collected with a NanoSight^*™*^ LM10 system equipped with a LM14 green (535 nm) laser module and a cooled Andor camera (Andor-DL-658-OEM). The localization noise was estimated by the covariance of the trajectory positions [22] was 0.181 μm.

#### Optical recording of fluorescent nanoparticles under conditions of constrained diffusion

Nanoporous diblock copolymer membranes were immersed in suspension of fluorescent polystyrene beads for 2-3 days. The membrane average pore size was 1*μ*m with a standard deviation of 0.25*μ*m. To visualize diffusing particles inside the membrane pores the samples were excited under a fluorescence microscope with a diode laser of 532 nm and an excitation energy of 25 W/cm^2^. Each movie is a sequence of 1000-2000 images. A detailed description of the method is available elsewhere [13].

### Image processing and trajectory acquisition

Image processing and tracking for the videos captured by DFM, CLSM or NTA were carried out with Fiji [23]. Before tracking, we removed the background from each video. Therefore, the minimum intensity for each pixel along the time axis was estimated because particle spots are markedly brighter than the background. Each pixel value in every frame was then divided by the corresponding estimated background pixel value. Finally, the trajectories were extracted by the Fiji plugin TrackMate [24]. Therefore, every candidate particle position in every frame was identified by the “find maxima detector” [25]. The find maxima detector detects all local maxima, rank them by intensity, and then applies a “region growing” algorithm with a user-specified tolerance, beginning with the highest intensity maxima. If a maximum lies within a region of previously detected maxima the former is discarded. The advantage of the procedure is that it does not assume a specific size of a spot in contrast to the Laplacian of Gaussian detector. Furthermore, it is more robust against noise. Once the candidate positions have been detected they are refined by estimating the subpixel accuracy with an iterative process. Therefore, a quadratic function is fitted to the local intensity values and the maximum value of the fitted function is calculated. The process may be repeated if the distance between the original position and the refined position exceeds 0.5. The refined positions are then connected to trajectories using the robust tracking algorithm from Jaqaman [26]. By optimization methods, Jaqaman first connected positions of consecutive frames, thus creating trajectory segments which were then combined to generate a complete trajectory. The optimization method used by Jaqaman tries to find the most likely set of particle trajectories over all frames.

### Trajectory classification and characterization

Classification is a subject of machine learning, a popular field of which is “supervised learning”. In supervised learning a classification model is trained by known data which consist of one or multiple features and at least two associated categories (or “classes”). The objective of supervised learning is to apply the trained model to unseen features to allocate them to the trained categories. In this section we will briefly introduce the employed classification model called “Random forests” and give a detailed description which features were used for training and, of course, for the classification of unseen data.

#### Random forests^*™*^

Random forests (RF) is an ensemble-based method which uses an ensemble of decision trees. It was invented by Breiman and Cutler [27] and combines bagging and random feature selection. Given a dataset with N elements and M features per element, bagging randomly chooses (with replacements) N elements from the dataset. During bagging, approximately one third of the data in a given dataset is not selected. The not selected elements form the out-of-bag (OOB) sample which is used for the further error estimation of the decision tree. For constructing the decision tree *m* ≪ *M* features are randomly selected. Typically 500 trees are trained using this procedure and the predictions of all trees are combined and converted into single prediction. This is done by a vote of each decision tree for the different prediction classes and then use the class with the most votes as final prediction. For this work we used the “randomForest” package implemented for the software environment R [28].

#### Trajectory features

For characterizing the trajectories we developed a new library called “TraJ” [29]. It is freely available under MIT license and implements various features and simulation routines. For our approach, we used the following nine normalized features: The anomalous exponent alpha, asymmetry, efficiency, fractal dimension, gaussianity, kurtosis, mean squared displacement ratio, straightness and trappedness.

**Exponent alpha:** Alpha is the exponent of the model given by Eq (3) fitted to the MSD values estimated by Eq (1). It shows values *α* ≈ 1 for *α* > 1 ND, for DM and *α* < 1 for AD and CD.

**Asymmetry:** As previously defined by Saxton [30] and extended by Helmuth et al. [18] the asymmetry *a* is derived from the “radius of gyration” tensor and is defined by

$$\begin{array}{c} a=-log\left(1-\frac{{\left(\lambda_{1}-\lambda_{2}\right)}^{2}}{2{\left(\lambda_{1}+\lambda_{2}\right)}^{2}}\right) \end{array}$$ (12)

Where *λ*_1_ and *λ*_2_ are the eigenvalues of the radius of gyration tensor ***T***:

$$\begin{array}{c} T=\left(\begin{array}{cc} \frac{1}{N}\sum_{j=1}^{N}{\left(x_{j}-\langle x\rangle \right)}^{2} & \frac{1}{N}\sum_{j=1}^{N}\left(x_{j}-\langle x\rangle \right)\left(y_{j}-\langle y\rangle \right)\\ \frac{1}{N}\sum_{j=1}^{N}\left(x_{j}-\langle x\rangle \right)\left(y_{j}-\langle y\rangle \right) & \frac{1}{N}\sum_{j=1}^{N}{\left(y_{j}-\langle y\rangle \right)}^{2} \end{array}\right) \end{array}$$ (13)

**Efficiency:** The efficiency *E* is a measure for the linearity of a trajectory and relates the squared net displacement to the sum of the squared displacements:

$$\begin{array}{c} E=\frac{{\left|x_{N-1}-x_{0}\right|}^{2}}{\left(N-1\right)\sum_{i=1}^{N-1}{\left|x_{i}-x_{i-1}\right|}^{2}} \end{array}$$ (14)

**Fractal dimension:** The fractal path dimension is defined by Katz and George [31] as

$$\begin{array}{c} D_{f}=\frac{log(N)}{log(NdL^{-1})} \end{array}$$ (15)

where L is the total length of the path, N is the total number of steps and d is the largest distance between any two positions. According to Katz and George the fractal dimension takes values around 1 for straight trajectories (e.g. DM), around 2 for random trajectories (e.g. ND) and around 3 for constrained trajectories (e.g. CD or AD).

**Gaussianity:** This feature is proposed by Ernst et al. [32] to characterize anomalous diffusion modes and is defined by

$$\begin{array}{c} g\left(n\right)=\frac{\left\langle r_{n}^{4}\right\rangle }{2{\left\langle r_{n}^{2}\right\rangle }^{2}} \end{array}$$ (16)

with

$$\begin{array}{c} \left\langle r_{n}^{4}\right\rangle =\frac{1}{N-n}\sum_{i=1}^{N-n}{\left|x_{i+n}-x_{i}\right|}^{4} \end{array}$$ (17)

ND shows value of zero because their increments have a Gaussian distribution while other diffusion modes show deviations of zero.

**Kurtosis:** To calculate the Kurtosis K we have projected the positions on the dominant eigenvector of the radius of gyration tensor [18]:

$$\begin{array}{c} K=\frac{1}{N}\sum_{i=1}^{N}\frac{{\left(x_{i}^{p}-\bar{x^{p}}\right)}^{4}}{\sigma_{x^{p}}^{4}} \end{array}$$ (18)

where *x*^*p*^ are the one dimensional projected positions, $\bar{x^{p}}$ the mean projected position and *σ*_*x*^*p*^_ the standard deviation of *x*^*p*^.

**Mean squared displacement ratio:** The mean squared displacement ratio characterizes the shape of the MSD curve and we define it as follows:

$$\begin{array}{c} {\left\langle r^{2}\right\rangle }_{n_{1},n_{2}}=\frac{\left\langle r_{n_{1}}^{2}\right\rangle }{\left\langle r_{n_{2}}^{2}\right\rangle }-\frac{n_{1}}{n_{2}} \end{array}$$ (19)

with *n*_1_ < *n*_2_. When inserting Eqs (2)–(5) into Eq (19) it is straightforward to follow that for ND it is 0, for AD and CD > 0 and for DM < 0.

**Straightness:** Similar to the efficiency *E* the straightness *S* of a trajectory relates the net displacement to the sum of step lengths:

$$\begin{array}{c} S=\frac{\left|x_{N-1}-x_{0}\right|}{\sum_{i=1}^{N-1}\left|x_{i}-x_{i-1}\right|} \end{array}$$ (20)

**Trappedness:** Following Saxton [30] the probability that a diffusing particle with diffusion coefficient *D* and traced for period of time *t* is trapped in a bounded region with radius *r*_0_ is given by:

$$\begin{array}{c} p_{t}=1-\exp\left(0.2048-0.25117\left(\frac{Dt}{r_{0}^{2}}\right)\right) \end{array}$$ (21)

We replaced *r*_0_ by the estimated half of maximum distance between any two positions and we replaced *D* by the estimated short-time diffusion coefficient. Short-time means, that we only used the first two time lags for estimation.

## Results

The simulation of trajectories enabled us to generate a set of artificial trajectories with well defined properties for all four motion types. The features of each trajectory and its class served as training data. The trained model was then evaluated by the OOB data and a sensitivity analysis was carried out to define the working range. Finally, the trained model will be applied to experimental trajectories from cell and other studies to demonstrate the usefulness of the method.

### Classification model training

For each motion type 5000 trajectories were simulated as training dataset. The timelag between two steps was set to Δ*t* = 1/30 which is a typical value in experimental setups and the diffusion coefficient was set to 9.02 *μ*m^2^/s which is a typical value of freely diffusing nanoparticle with a diameter of 50 nm in water at 22°C. Other parameters of each motion type were chosen randomly: The number of steps for each trajectory were selected between 30 and 600 steps. To get statistically meaningful features the trajectory should contain at least 30 positions. However, the trajectory features converged against a stable value for more available positions so it was safe to set the upper bound to 600 steps. For the simulation of confined diffusion we chose the boundedness randomly between 1 and 6. The lower bound of 1 was chosen because boundedness values <1 means that the average trajectory was not influenced by the confinement. To our experience, values larger than the upper bound of 6 do not influence the trajectory features. The radius corresponding to a given boundedness was then derived by Eq (9). The anomalous exponent for AD simulation was chosen between 0.3 and 0.7 which is the typically observed range [14, 33, 34]. For active transport the directed motion factor R was selected between 1 and 17. A low R value represents trajectories with a strong diffusion component relative to the active component. Notably, the higher the R value becomes, the stronger is it influenced by the active transport component. The corresponding velocity for the given diffusion coefficient was derived by Eq (11). To simulate localization noise, we added normal Gaussian noise with zero mean and standard deviation *σ* to each position. Therefore we define two signal to noise ratios (SNR). The first one was used for ND, AD and CD and is defined as follows:

$$\begin{array}{c} SNR_{1}=\frac{\sqrt{D\Delta t}}{\sigma } \end{array}$$ (22)

Because DM is modeled with a normal diffusion and an active component we define it as:

$$\begin{array}{c} SNR_{2}=\frac{\sqrt{D\Delta t+v^{2}\Delta t^{2}}}{\sigma } \end{array}$$ (23)

Both SNR values were randomly set between 1 and 9. The source code for the generating the training data is freely available [35].

### Benchmarks

The performance of a random forest classifier is typically characterized by the out-of-bag (OOB) error-rate. Each element in the dataset is contained in around 1/3 OOB samples of the constructed trees. The proportion of wrong classifications given by these third of trees averaged over all elements is the OOB error-rate which amounts to 10.6% for directed motion, 1.9% for anomalous-, 5.5% for confined- and 10.6% for normal diffusion. To get an impression which classes are mistaken, Table 1 shows the confusion matrix.

**Table 1:** Confusion matrix based on out-of-bag data.

		Prediction			
		Directed	Anomalous	Confined	Normal
Reference	Directed	89.44%	0%	0.08%	10.48%
	Anomalous	0%	98.1%	1.34%	0.56%
	Confined	0%	2.88%	94.5%	2.62%
	Normal	6.36%	1.08%	3.14%	89.42%

Furthermore, a 10-fold cross validation (CV) for the training dataset gave a kappa value of 0.89 and an accuracy of 0.92. The sensitivity / specificity values for different types of motion evaluated by CV are shown in Table 2.

**Table 2:** Sensitivity and specificity for different motion types evaluated by 10-fold cross validation.

	Sensitivity	Specificity
Directed	0.89	0.98
Anomalous	0.98	0.98
Confined	0.93	0.98
Normal	0.89	0.95

### Feature importance

**Fig 2:**
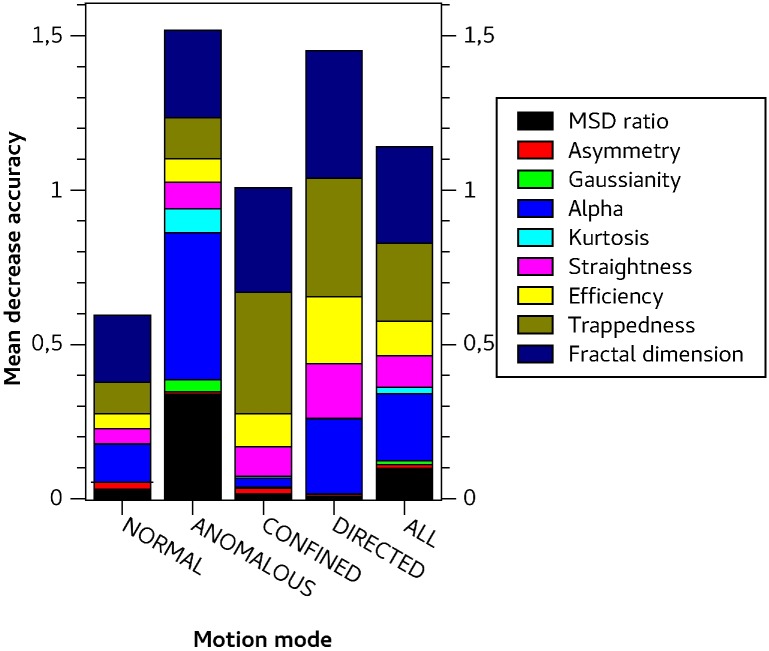
Class-specific mean decrease in accuracy (MDA) for directed motion and normal-, anomalous- and confined-diffusion. The outer right column shows the MDA over all classes.

Besides general benchmarks we evaluated the importance of each feature for every class. Therefore, the OOB error was estimated, then the values for a single feature were permuted and the OOB error was calculated again. The average of the differences is the mean decrease in accuracy for that feature and is shown in Fig 2.

### Classification and segmentation

For the classification process we divided each trajectory in overlapping sub-trajectories with a window length *ω*. That means, for a trajectory with *N* positions we generated *K* = *N* − *ω* + 1 sub-trajectories. After this step, each of the *K* sub-trajectories could optionally be resampled: Instead of using each position only every *i*-th position was used where *i* = 1 means no re-sampling. This procedure increases the SNR because it increases the time-lag between successive positions. However, the higher the resampling rate was, the lower became the number of used positions in each sub-trajectory which decreased the precision of feature estimation. Therefore the re-sampling rate was chosen in such a way that the number of positions in the resampled trajectory was at least larger by 30. After resampling we applied the random forest classification on each of these *K* sub-trajectories and got the result class *c* and the confidence probability *p*_*c*_ for *c*. Following and extending the voting procedure of Helmuth et al. [18], each position of the trajectory, which was contained in the sub-trajectory, received a vote for *c*. We used a weight which equals the probablitity *p*_*c*_ instead of a unit weight. In case of a resampled trajectory, positions which were omitted due to re-sampling are voted as well. Finally, we labeled each position with the class which got the majority of votes. The average of the vote weights for a given position was taken as confidence level for that position. Successive positions with the same class were then combined into a single trajectory segment. The average from the confidence levels of each position belonging to this segment was taken as the confidence level for that segment.

### Sensitivity analysis

**Fig 3:**
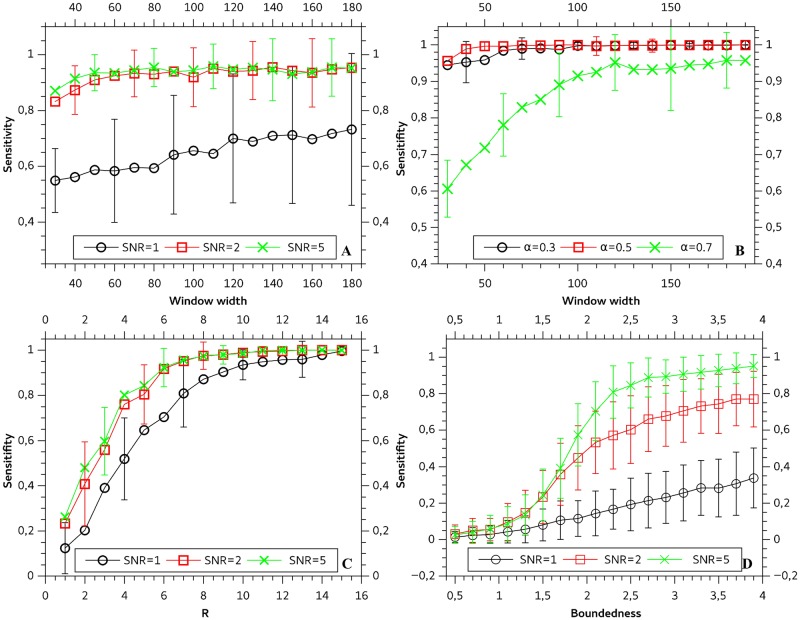
Sensitivity measurements for normal diffusion (A), anomalous diffusion (B), directed motion (C) and confined diffusion (D).

When applying a classification routine on experimental data it is important to know how the experimental boundary conditions influence the classification quality. Besides the SNR, the diffusion process itself (R for DM and the boundedness for CD) and the used window length have an impact on the classification quality. Fig 3 shows these dependencies for all motion types. Whereas ND (Fig 3A) is only slightly depending on the window length, it is strongly influenced by the SNR. However, with higher SNR the sensitivity for ND converges rapidly to values > 0.8. The sensitivity for the AD (Fig 3B) depends mainly on the anomalous exponent alpha and the window length. Large alpha values lead to misclassification with ND because the ND can be interpreted as AD with *α* = 1. With regard to AD we only show data for SNR = 5, because localization noise leads to an artificial appearance of AD [36] which contrary to expectations increases the sensitivity. Active transport (Fig 3C) shows only a weak dependence of SNR but it is strongly dependent on the parameter R. For R values larger than 6 it shows a sensitivity > 0.7 even for low SNRs and converges towards 1 for larger R values. The boundedness and SNR has a high impact on the classification sensitivity of confined diffusion (Fig 3D). Whereas for a SNR of 1 the sensitivity has a maximum value 0.42 even for high boundedness, it expeditiously improves for higher SNR.

### Application of the classifier to experimental data

To demonstrate its applicability, we applied the proposed classifier to trajectories of freely diffusing particles recorded in a NTA video, to constrained diffusing particles recorded in a FM video and to more heterogeneous trajectories of diffusing particles in V79 cells recorded in a DFM and CLSM video. To obtain meaningful results a judicious choice of the classification parameters is mandatory. One parameter is the window length which is always a tradeoff between statistical accuracy and time resolution. Most importantly, the SNR has a strong impact on results. Unfortunately the SNR is often unknown under experimental conditions and may furthermore differ between the particles. However, as described in section “Classification and segmentation” the signal to noise ratio could be increased by re-sampling the trajectory. We classified the trajectories using a window length of 120 and then applied the following procedure to find a reasonable re-sampling rate: We calculated the histogram *H*_*i*_(*c*_*k*_) of trajectory positions with class *c*_*k*_ resampled with the resampling rate *i*. According to Cao and Petzold [37] the difference between two histograms could be measured by

$$\begin{array}{c} \Delta H\left(i\right)=\sum_{k=1}^{N}\frac{\left|H_{i}\left(c_{k}\right)-H_{i+1}\left(c_{k}\right)\right|}{N_{c}} \end{array}$$ (24)

where *N*_*c*_ is the number of classes. We then choose the resampling rate *i*, with the lowest Δ*H*. According to Fig 3 the SNR looses its impact at a certain level which leads to low Δ*H*.

**Fig 4:**
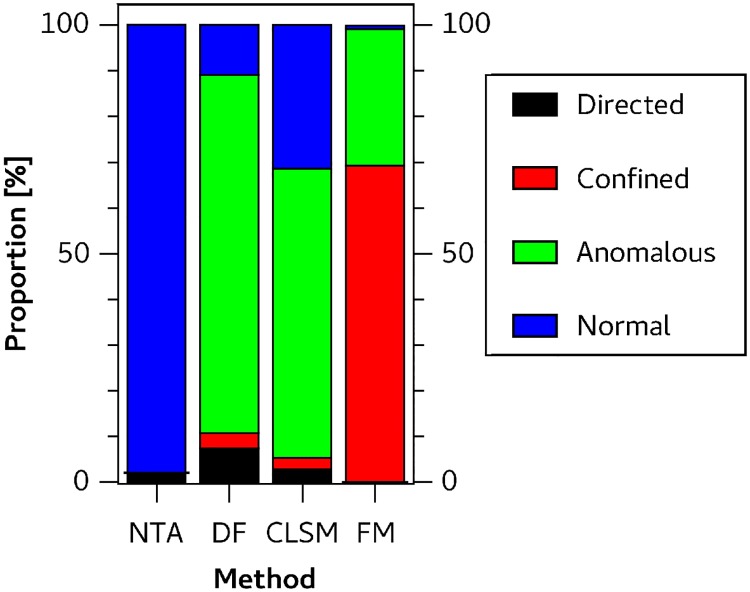
Motion class proportions of classified positions of experimental trajectories captured by NanoSight NTA (N = 334), darkfield microscopy (N = 90), confocal laser scanning microscopy (N = 41) and fluorescence microscopy (N = 28).

For our experimental setups the calculated Δ*H* is listed in Table 3, which suggests that resampling is unnecessary if trajectories were obtained with NTA. This appears plausible because NTA measurements have an experimentally measured localization noise value of approximatly 0.181 μm and a diffusion coefficient of 4.51 μm^2^ / s which results in a SNR of 2.14, which is sufficient to give stable results. For the FM, DFM and CLSM setup we used a resampling rate of 3 according to Table 3. After the appropriate resample rate was identified we re-analyzed the trajectories with a window length of 90 to improve time resolution. Fig 4 shows the motion class proportions for the different experimental setups.

**Table 3:** Histogram distances for different resampling rates. The lowest distance for each method is highlighted in bold.

		Resampling rate		
		i = 1	i = 2	i = 3
Method	NTA	**0.004**	0.05	0.016
	DFM	0.039	0.025	**0.017**
	CLSM	0.061	0.045	**0.017**
	FM	0.078	0.039	**0.003**

#### Nanoparticle Tracking Analysis

**Fig 5:**
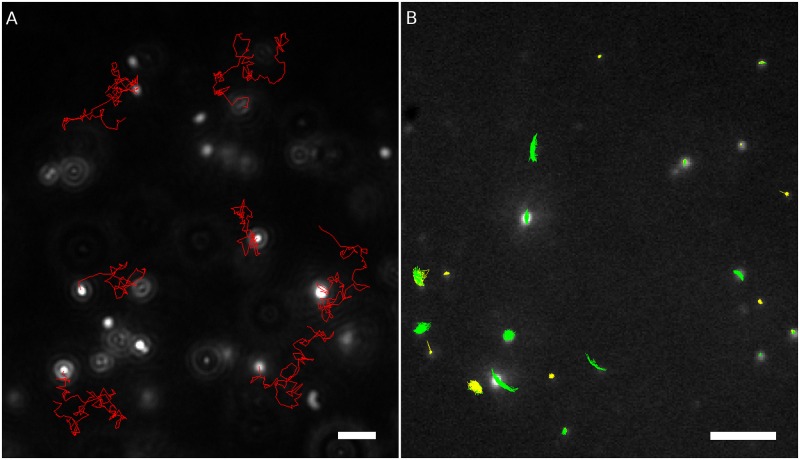
Images captured with (A) NTA device and (B) fluorescence microscopy with overlayed trajectories recorded from the whole video sequence. Red trajectories were classified as normal diffusion, green trajectories were classified as anomalous diffusion, yellow trajectories as confined diffusion. Bar: 5 μm.

Analysis of trajectories obtained with NTA device showed that 98% of the trajectory positions were classified as ND. Fig 5A shows six typical trajectories. The mean number of segments per trajectory is 1.14, which means that most of trajectories are composed of a single ND segment. A ND segment had a mean duration of 10.9s and an active transport segment has a mean duration of 3.6 s. The mean diffusion coefficient was 3.87 μm^2^/s.

#### Constrained diffusion of fluorescent nanoparticles

Approximately 70% of the trajectory positions measured by fluorescence microscopy were classified as CD and almost 30% as AD. An insignificant fraction of 0.75% were identified as ND. The mean estimated confinement size was 0.389 μm. Fig 5B shows representative trajectories.

#### Darkfield microscopy

**Fig 6:**
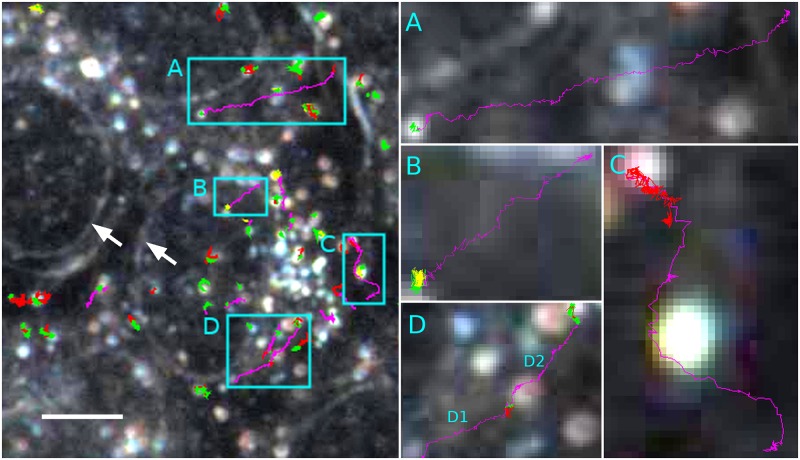
NP trajectories in live V79 fibroblasts as classified by the program. Several cells are gathered within the field of view (left image). While cell borders are not visible in this focal plane, nuclear envelopes stand out clearly under darkfield illumination (white arrows). A total of 246 particle trajectories was identified as either normal diffusion (red), confined diffusion (yellow), anomalous diffusion (green) or directed motion (magenta). Boxed areas (A-D) show selected cases of directed motion. The time durations of the directed motion were 14.6s (A), 7.4s (B), 10s (C), 6.4s (D1), 10.8s (D2). Note that directed motion ends up with confined or anomalous diffusion (A, B, D) or emanates from a phase of free diffusion (C). Bar: 5 μm.

The trajectories measured with DFM showed heterogeneous motion types. Around 10.9% were classified as ND, 78.4% as AD, 3.4% as CD and 7.3% as DM. Fig 6 shows representative trajectories. Following Eqs (2)–(5) the different motion types were characterized as follows: The mean diffusion coefficient for ND classified segments was 0.05 μm^2^ / s, for AD we obtained a mean diffusion coefficient 0.013 μm^2^ / s and mean anomalous exponent *α* = 0.22. When segments were classified as confined diffusion we calculated a mean diffusion coefficient of 0.436μm^2^/s and a mean confinement radius *r*_*c*_ = 0.366 μm. Mean shape parameters were *A*_1_ = 0.396 and *A*_2_ = 0.632. Actively transported particles had an estimated mean velocity of 0.511μm/s and a mean diffusion coefficient of 0.077 μm^2^ /s

#### Confocal laser scanning microscopy

**Fig 7:**
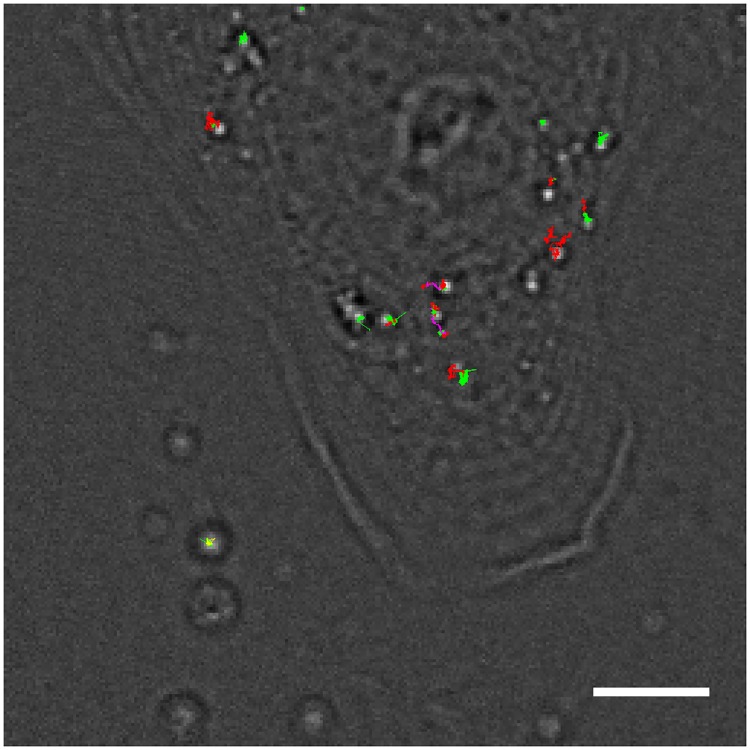
Image from confocal laser scanning microscopy overlayed with particle trajectories recorded from the whole video sequence. Red trajectories were classified as normal diffusion, yellow as confined diffusion, green as anomalous diffusion and magenta as directed motion.

The motion type proportions of CLSM video data were similar to those from DFM video data. Fig 7 shows classified trajectories. From 107 classified segments 60 were classified as AD with a mean anomalous exponent *α* = 0.29 and diffusion coefficient of 0.003 μm^2^/ s. Another 37 segments are classified as ND with a mean diffusion coefficient of 0.005 μm^2^/s. Only 6 segments are identified as DM with a mean diffusion coefficient of 0.006 μm^2^ /s and mean velocity of 0.119 μm/s. CD was observed for 4 segments. The mean confinement radius was 0.117 μm and the mean shape parameters amounted to *A*_1_ = 0.370 and *A*_2_ = 1.937.

### TraJclassifier Fiji plugin

The proposed method was implemented as an open source (MIT license) Fiji plugin called TraJClassifier. Before the classification starts, the user has to select the trajectory xml file exported by TrackMate. Thereafter the minimum tracklength, the window size and some system specific parameters need to be specified. Internally, the classification is carried out by the randomForest R package [38] accessed by the Renjin interpreter (http://www.renjin.org/). When the plugin has finished the classification process it shows original video data overlayed with the classified trajectories, as shown in Figs 5–7. Furthermore, five results tables will be opened, one for each motion type with an row for each trajectory segment with the according motion type. Futhermore. each row contains the estimated features, the fitted MSD curve parameters, a confidence level, unique identifier consisting of a sub-trajectory ID and a parent trajectory ID. This IDs could be overlayed with the original video data as well. The fifth table contains a summary of the parent trajectories.

## Discussion & Conclusion

In this work we present a novel method for the automated classification and segmentation of particle trajectories which is applicable to various kinds of single particle tracking experiments. While the method was based on several features from previous single particle tracking analyses, the parameters “fractal dimension” and “trappedness” were included into the automated classification and segmentation of diffusion trajectories the first time. As both features together are responsible for approximately 55% of the achieved accuracy a fairly improved allocation of motion types to diffusion trajectories is to be expected. Moreover, to the best of our knowledge, this is the first classification approach which was validated by positive controls instead of using simulated data only. A sensitivity analysis defined the working range of the classification method and may be combined with a systematic procedure to modify and optimize the SNR by re-sampling the trajectory. Besides dividing a trajectory into several segments with distinct motion types it estimates the confidence of the classification for that segment. The evaluation of the program with simulated data revealed a considerable accuracy of 0.92. However, acceptable accuracy can be achieved only if the experimental boundary conditions are sufficiently good. Ideally, the SNR should be at least 2. The procedure to increase the SNR (see section “Application of the classifier to experimental data”) is a trade-off between statistical accuracy of the feature estimates and the SNR. Recently published results give additional advice as to how experimental parameters can be optimized to improve precision [39]. To demonstrate the applicability and versatility of the method the approach was applied to image series obtained with different experimental light microscopic setups. Among these the NTA method undoubtedly serves as a source for unrestrained ND, as there are no restrictions for diffusing particles. In line with this the classification results identified more than 98% of NTA trajectory positions as ND. Unfortunately, there is no established positive control for constrained diffusion like CD and AD at the moment [40]. However, a candidate model with particles trapped in circular containments was developed by Haramagatti et al. [13] and is discussed by Saxton [40]. We applied our procedure to footage provided by Haramagatti et al. and got plausible results. Due to the geometry of the confinements it was not surprising that nearly all trajectories were classified as constrained diffusion type. However, the calculated mean confinement size of 0.389 μm differed from the 1 μm diameter observed by scanning electron microscopy (SEM). This was observed by Haramagatti et al. as well and was explained by a combination of hydrodynamic hinderance due to wall effects, repulsive forces and a reduction in confinement size by decorating the walls with polymer chains. In any case, results for intracellular trajectories appear highly plausible because the majority of intracellular trajectories were classified as AD, which is in accordance with other authors (a recent review is given by Höfling and Franosch [8]). Moreover cases of directed motion were obviously classified correctly. However, the number of AD trajectories tends to be overestimated because localization noise adds apparent AD to the trajectories [36]. This is especially a problem for particles which are bound to cellular structure and therefore appear immobile. Monte Carlo simulation of trajectories made out of 60 positions of pure standard normal noise showed that 95% have alpha values smaller than 0.06. Such simulations could be a way to reclassify questionable trajectories but need further investigations.

An unexpected finding was the occurrence of ND in cells, which was found in 10% or 30% of trajectories obtained with DFM or CLSM, respectively. Also mean velocities and confinement radii differed for DFM and CLSM trajectories. The different mean velocities may be explained by the fact that the trajectories recorded by CLSM were exclusively from gold NP inside the cell whereas in DFM particles are recorded from a thicker optical plane inside and outside the cells and, therefore, could be followed for a longer period. Consequently, DFM revealed more complex patterns of particle motion with longer phases of directed transport, as shown in Fig 6. However, as no image stacks were evaluated, a clear cut decision could not be made as to whether the observed directed motion of a gold NP occurred inside a cell or reflects its sliding on the outside of the cell membrane. Since some particles seemingly crossed the border of (the underlying) nuclear envelope without delay and because the 50 nm large nanoparticles used in this study are too large to pass the pores of the nuclear envelope, the latter assumption is far more likely. In general, the nuclear pore complex may allow for a passage of nanoparticles smaller than 5 nm such as fluorescent fullerenes [41]. These processes should be observable with well adapted forms of laser scanning microscopy and it may be challenging to analyze with the program the type of diffusion through a nuclear pore. However, such observations cannot be made with the current DFM technique which is limited to light scattering nanoparticles of at least 30 nm size. Assuming that directed motion observed here occurs on the cell surface is furthermore in line with what Wang and co-workers [42] described using a three dimensional DFM approach. These authors were the first who described that nanoparticles may travel along the outer membrane toward the upper pole of the cell. The driving force underlying this particle transport is still unknown but resembles a phenomenon which has long been known as cap formation [43]. Mechanistically it may involve a binding of NP to integrins traversing the membrane and being moved by intracellular actin filaments [44]. Further studies unravelling these processes may now take advantage of the software tool presented here. In some cases trajectories of direct motion eventually turned into anomalous or confined forms of diffusion. The study of Wang et al. [42] suggests that NP demonstrating these forms of movement were finally trapped in endocytotic vesicles which was also found by electron microscopy for the same type of gold NP (own unpublished results). Thus, all forms of motion observed with DFM and confocal microscopy are in line with the study of Wang et al. [42] or with electron microscopy results. However, and with respect to the latter, they shed far more light on the dynamic processes of adhesion, internalisation and handling of nanoparticles by live cells.

In summary, our approach provides a practical and device-independent method to classify and segment particle trajectories into four main motion types. We demonstrated the applicability of the program for different experimental setups and synthetic trajectories. An extensive report characterizes each segment of a trajectory by its basic motion type specific parameters and other features. We believe that the proposed tool may be helpful to gain insights into biological processes underlying the dynamics of nanoparticles inside cells.
